# Expression of CDK9 in endometrial cancer tissues and its effect on the proliferation of HEC-1B

**DOI:** 10.1515/biol-2021-0136

**Published:** 2021-12-31

**Authors:** Wen Yang, Shaoyan Liu, Qiuping Luo, Xuexian Tan

**Affiliations:** Department of Pathology, The Third Affiliated Hospital, Guangzhou Medical University, 510150 Guangzhou, China; Key Laboratory for Major Obstetric Diseases of Guangdong Province, The Third Affiliated Hospital, Guangzhou Medical University, 510150 Guangzhou, China

**Keywords:** endometrial cancer, CDK9, clinicopathology, the prognosis, cell proliferation

## Abstract

Endometrial cancer (EC) is one of the most common malignant tumors in the female reproductive system, which has been threatening the life and health of many women. Its incidence and mortality rate remain high, resulting in a low survival rate. In this study, the expression of cyclin-dependent kinase 9 (CDK9) in EC tissues was investigated, and its effect on the proliferation of EC cell line HEC-1B was preliminarily analyzed. RT-qPCR and Western blotting showed that CDK9 mRNA and protein were significantly reduced in the small interfering (si)RNA interference group compared with the siRNA control and blank control groups. MTT assay showed that the EC cell proliferative ability was significantly decreased, and phosphorylated-phosphatidylinositol 3 kinase (p-PI3K)/PI3K and phosphorylated-protein kinase B (p-AKT)/AKT were significantly reduced in the siRNA interference group compared with the siRNA control and blank control groups. In conclusion, the high expression of CDK9 is a factor of poor prognosis in EC, and the reduction of CDK9 can inhibit HEC-1B cell proliferation, which may be related to the inhibition on the activation of the PI3K/AKT signaling pathway.

## 1 Introduction

Endometrial cancer (EC) is a common malignancy of the female reproductive system, ranking eighth among malignancies in China. The common symptoms are mainly vaginal bleeding and menstrual disorder and mostly occur in perimenopausal and postmenopausal middle-aged women. However, there is a trend toward younger patients being diagnosed. The main reason lies not only in risk factors, such as obesity and diabetes, but also in the area where patients live. Because EC is not evident in the early stages, patients often progress to advanced stages at the time of diagnosis [1]. Its prognosis is poor, with a 5 year survival rate <40% [2,3]. Therefore, understanding the molecular mechanism of EC is very important for improving diagnosis and treatment. If appropriate countermeasures are taken according to its molecular mechanism, the survival cycle of patients will be greatly improved, and the mortality of EC will be reduced. Cyclin-dependent kinase 9 (CDK9) is a serine/threonine protein kinase that activates RNA polymerase I to promote transcription and participate in cellular physiological and biochemical processes [4]. CDK9 plays an important role in maintaining transcription homeostasis. Recent studies have shown that CDK9 plays an oncogenic role in various malignant tumors such as osteosarcoma, colon cancer, and glioblastoma [5,6,7]. CDK9 is increasingly regarded as a major therapeutic target for developing antitumor drugs due to its important value in tumor growth and the occurrence of virus infection in patients. In addition, researchers found that CDK9’s role is focused on intracellular regulation and not primarily in the cell cycle, as previously suspected [8]. However, the expression and role of CDK9 in EC are still unclear, creating a need for further analyses and discussion to determine the mechanisms of action arising from its effects and developmental patterns. In this study, the expression of CDK9 in EC patients was detected, and its relationship with clinicopathology and prognosis was analyzed. Furthermore, the effect of CDK9 on the proliferation of EC cells and its mechanism were further explored to find an effective treatment for the treatment and remission of EC.

## 2 Materials and methods

### 2.1 General data

A total of 62 cases of EC, with an average age of 54.79 ± 2.78 years, were diagnosed by histological examination, who were without other malignant tumors, preoperative radiotherapy, chemotherapy, or hormone therapy. During the same period, 34 patients diagnosed with atypical endometrial hyperplasia (mean age of 54.91 ± 3.39 years) and myoma of the uterus underwent total hysterectomy were selected. Postoperative pathological examination confirmed normal endometrium in 22 patients with normal endometrium tissue (mean age of 54.77 ± 3.46 years). The overall survival of EC patients ranges from the beginning of surgery to the end of follow-up. The shortest overall survival of EC patients was 12 months, and the longest follow-up date was 60 months [9].


**Informed consent:** Informed consent has been obtained from all individuals included in this study.
**Ethical approval:** The research related to human use has been complied with all the relevant national regulations, institutional policies, and in accordance with the tenets of the Helsinki Declaration and has been approved by the Committee on Ethics of the Third Affiliated Hospital of Guangzhou Medical University.

### 2.2 Reagents

HEC-1B cell line was purchased from Shanghai Xin Yu Biotech Co., Ltd (Shanghai, China). Total RNA extraction reagent, RT-qPCR kit (SYBR Green), and primers were purchased from TaKaRa Bio-engineering (Dalian) Co., Ltd (Liaoning, China). A ready-to-use immunohistochemical UltraSensitive TM SP detection kit was purchased from North Fuzhou Maixin Biotechnology Development Co., Ltd (Fuzhou, China). The interference control vector, CDK9 interference vector, and the corresponding packaging plasmid were all designed and constructed by Guangzhou RiboBio Co., Ltd (Guangdong, China). HRP-labeled rabbit secondary antibodies and rabbit antihuman CDK9, p-PI3K, p-AKT, phosphatidylinositol 3 kinase (PI3K), protein kinase B (AKT), and glyceraldehyde-3-phosphate dehydrogenase (GAPDH) monoclonal antibodies were purchased from Abcam Company (Cambridge, United Kingdom). Lipofectamine TM2000 was purchased from Thermo Fisher Inc., USA (Carlsbad, CA, USA).

### 2.3 Immunohistochemical assay

The paraffin-embedded tissue was fixed with 10% formalin [10], and slices with a thickness of 5 μm were prepared. The slices were dewaxed and rehydrated in xylene and then added with citric acid buffer (pH 6.0). After heating in the microwave oven, 3% H_2_O_2_ was dropped for 10 min. CDK9 antibody (1:50) was added and incubated overnight at 4°C. The test was performed using the ready-to-use immunohistochemical UltraSensitive TM SP test kit. Following DAB staining, hematoxylin redyeing, and neutral resin seal, the material was observed under the microscope, and the image was obtained.

### 2.4 Cell culture, transfection, and groups

HEC-1B cells were cultured in Roswell Park Memorial Institute (RPMI)-1640 medium (10% fetal bovine serum and 0.01 mg/mL insulin) at a density of 1 × 10^5^/mL. HEC-1B cells were divided into the siRNA interference group, the control siRNA group, and the blank normal control group. The specific transfection was performed by Lipofectamine TM2000 as follows: Packaged plasmids 1, 2, and CDK9 interference vector were added into the opti-MEM at a 5:3:2 ratio, mixed, then stood for 20 min, mixed with PE transfection reagent, and slowly added into HEC-1B cells. The incubation time was 48 h, and cells were collected.

### 2.5 Real time-quantitative polymerase chain reaction (RT-qPCR) experiment

RNA was extracted from cells using the total RNA extraction reagent, complementary DNA was synthesized with a reverse transcription kit, and CDK9 mRNA expression was detected with a real-time quantitative PCR kit, using β-actin as an internal reference. CDK9 forward: 5′-AAAACGAGAAGGGGGTTCC-3′, reverse: 5′-CCTTGCAGCGGTTATAGGGG-3′ and β-actin forward: 5′-ACTCTTCCAGCCTTCCTTC-3′, β-actin reverse: 5′-GATGTCCACACGTCACACTTC-3′. Reaction system: 2× Master Mix 10 µL, 0.3 µL forward primer (10 µmol/L), 0.3 µL reverse primer (10 µmol/L), Template 1 ng, double steamed water were added to 20 µL. Reaction procedure: 95°C for 10 min, 1 cycle; 95°C for 10 s, 60°C for 30 s, for a total of 40 cycles. The RT-qPCR instrument automatically generates the melting curve. The relative quantitative analysis was carried out by 2^−ΔΔCt^ method.

### 2.6 3-(4,5)-dimethylthiahiazo (-z-y1)-3,5-di- phenytetrazoliumromide (MTT) assay

Cells were inoculated in 96-well plates with 2 × 10^3^ cells per well for 24 h. A total of 10 μL of MTT solution was added to each well and incubated for another 4 h. The supernatant was discarded, and dimethylsulfoxide was added for full dissolution. The absorbance of each well was detected at 490 nm, and the cell proliferation rate was calculated. Proliferation rate (%) = (OD value of experimental group − OD value of background group)/(OD value of normal control − OD value of background group) × 100%.

### 2.7 Colony formation assay

Cells were inoculated with 500 cells per well in six-well culture plates for 10 days, and the supernatant was discarded when visible clones appeared. It was cleaned twice by PBS, then added 1 mL of 40 g/L paraformaldehyde fixative, and stood for 15–20 min. The supernatant was discarded, cleaned twice by PBS, added with 1 mL of crystal violet staining solution, and stood for 15–20 min. After carefully washed with tap water and dried naturally, the culture plates were scanned for photos, and the number of clones visible was counted.

### 2.8 Western blot assay

Cells were added with radioimmune precipitation assay lysis buffer to extract total protein. The same concentration of protein was taken for sodium dodecyl sulfate polyacrylamide gel electrophoresis, then the membrane was transferred, and the primary antibodies GAPDH (1:10,000), CDK9 (1:1000), p-PI3K (1:1,000), p-AKT (1:5,000), and AKT (1:2,000) were added, and incubated overnight. After soaking with the secondary antibody (1:20,000) at room temperature for 3 h, the membrane was cleaned, electro-chemi-luminescence was performed, and pictures were taken using a chemiluminescence imaging system.

### 2.9 Statistical analysis

All experimental data were analyzed by SPSS 21.0 soft-ware (IBM SPSS, USA). The measurement data were expressed as (*x* ± *s*). One-way analysis of variance was used for comparison among groups. Enumeration data were expressed as (%), *χ* ^2^/Fisher test. Kaplan–Meier survival analysis were used for comparison of intergroup rates. Test level was set as *α* = 0.05 (double-tailed).

## 3 Results

### 3.1 CDK9 expression in tissues from EC patients

**Figure 1:**
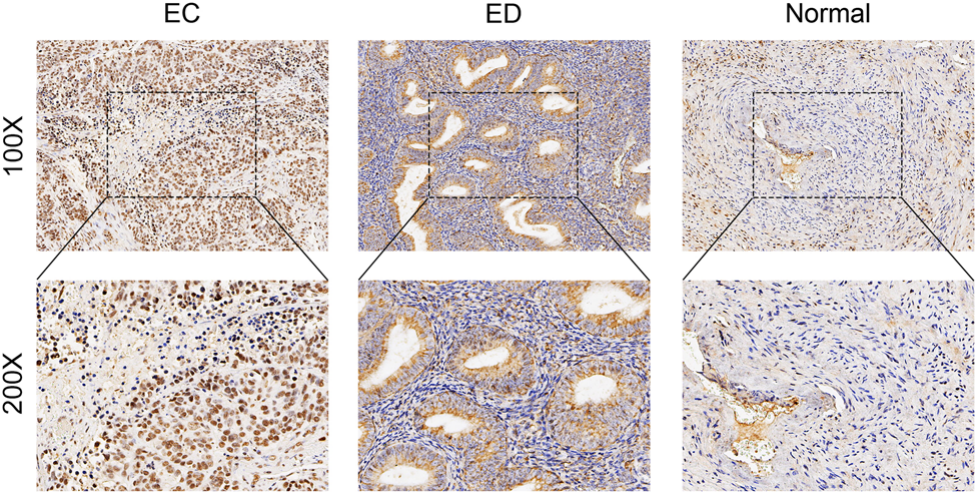
Immunohistochemistry assay of CDK9 expression. The immunohistochemistry assay on microscopic sections from EC tissues, endometrial atypical hyperplasia, and normal endometrial tissue.

Immunohistochemical staining showed that the positive expression rate of CDK9 in tissues from EC patients (72.58%, 45/62) was significantly higher than that from patients with endometrial dysplasia (8.82%, 3/34) (*χ* ^2^ = 35.704, *p* = 0.000) and from normal endometrial tissue (4.55%, 1/22) (*χ* ^2^ = 30.340, *p* = 0.000). The result of immunohistochemical staining is shown in Figure 1.

### 3.2 Correlation between CDK9 expression and clinicopathological characteristics in EC patients

CDK9 expression was correlated with histological grade (*p* = 0.000), International Federation of Gynecology and Obstetrics (FIGO) stage (*p* = 0.000), depth of muscular invasion (*p* = 0.034), and lymph node metastasis (*p* = 0.000) but showed no significant association with pathological type (*p* = 0.985), as shown in Table 1.

**Table 1:** Correlation between CDK9 expression and clinicopathological characteristics in EC patients

Parameter	*n*	CDK9 Protein expression		*χ* ^2^/Fisher value	*p* value
		Negative	Positive		
FIGO staging				28.134	0.000
I Period	12	10	2		
II Period	31	1	30		
III–IV Period	19	6	13		
The depth of muscular invasion				4.502	0.034
≥1/2	20	2	18		
<1/2	42	15	27		
Lymph node metastasis				17.547	0.000
Exist	34	2	32		
Without	28	15	13		
Pathological pattern				0.000	0.985
Endometrioid adenocarcinoma	40	11	29		
UPSC	22	6	16		
Histopathological grading				15.393	0.000
G1	15	10	5		
G2	23	3	20		
G3	24	4	20		

### 3.3 Relationship between CDK9 expression and EC prognosis

**Figure 2:**
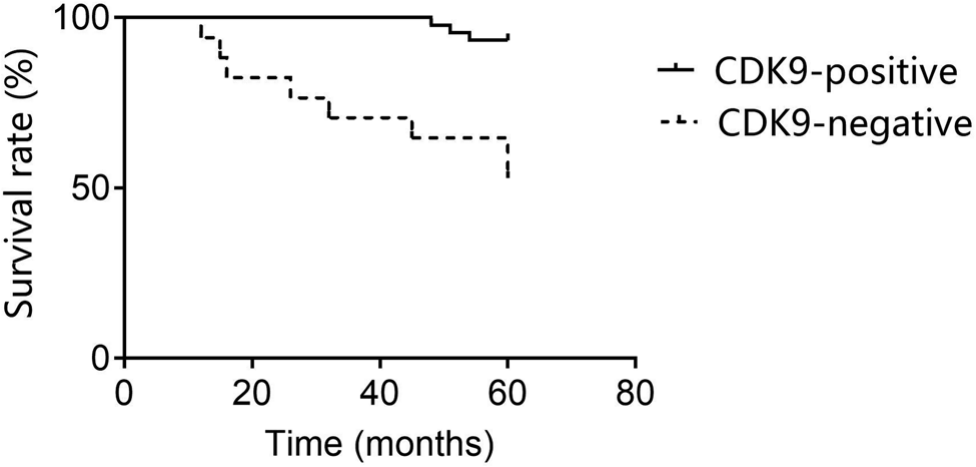
Relationship between CDK9 level and EC prognosis. Kaplan–Meier analysis showed the overall survival rate of EC patients with CDK9 positive and CDK9 negative expression. *p* < 0.05 represents a statistically significant difference.

According to the Kaplan–Meier analysis, the 5 year survival rate of EC patients with CDK9 positive expression was higher than that of patients with CDK9 negative expression (*χ* ^2^ = 16.04, *p* <0.05), as shown in Figure 2.

### 3.4 Interference of CDK9 mRNA and protein expressions in HEC-1B cells

**Figure 3:**
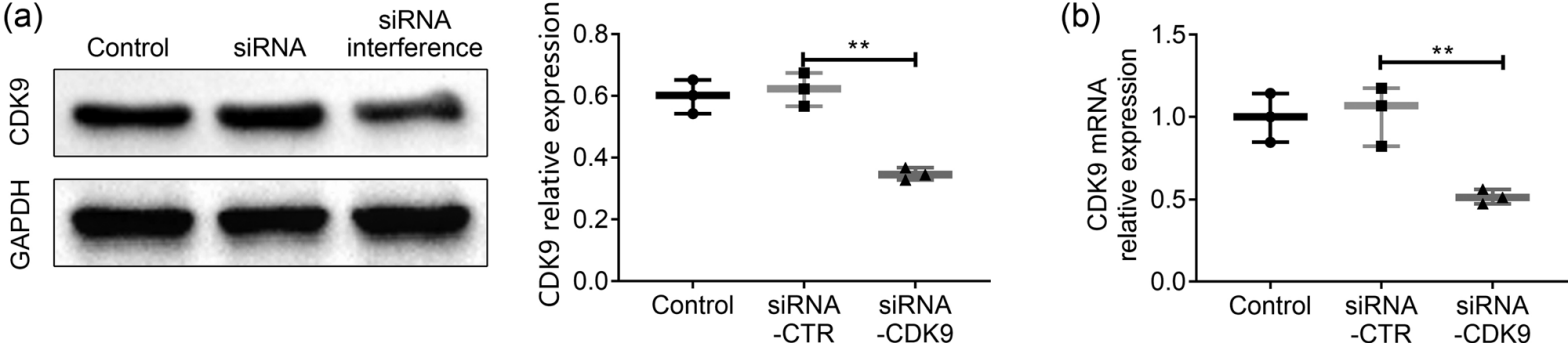
Interference of CDK9 mRNA and protein expressions in HEC-1B cells. (a) Left: Western blot assay was used to analyze the expression of CDK9 protein in HEC-1B cells. Right: the statistical analysis on Western blot assay. (b) The relative mRNA expression level of CDK9 in HEC-1B cells was determined by RT-qPCR assay. Data were expressed as mean ± SD. **p < 0.01 represents a statistically significant difference.

The results of RT-qPCR and Western blot assays showed that compared with the siRNA-CTR and siRNA-CDK9 groups, the mRNA and protein expressions of CDK9 in HEC-1B cells were significantly decreased by siRNA interference (*p* <0.01), as shown in Figure 3.

### 3.5 Effects of CDK9 interference on HEC-1B cell proliferation

**Figure 4:**
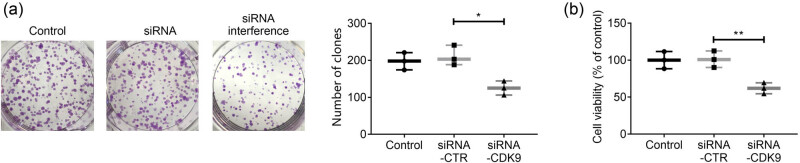
Effects of CDK9 interference on HEC-1B cell proliferation. (a) The plate colony formation experiment was used to detect the number of colony formation in HEC-1B cells. (b) The viability of HEC-1B cells was detected by the MTT method. Data were expressed as mean ± SD. *p < 0.05 and **p < 0.01 represent a statistically significant difference.

The results from MTT test and plate colony formation test showed that compared with the siRNA-CTR and siRNA-CDK9 groups, the proliferative ability of HEC-1B cells in the siRNA interference group was significantly decreased (*p* < 0.05 and <0.01, respectively), as shown in Figure 4.

### 3.6 Effects of CDK9 interference on PI3K/AKT signaling pathway in HEC-1B cells

**Figure 5:**
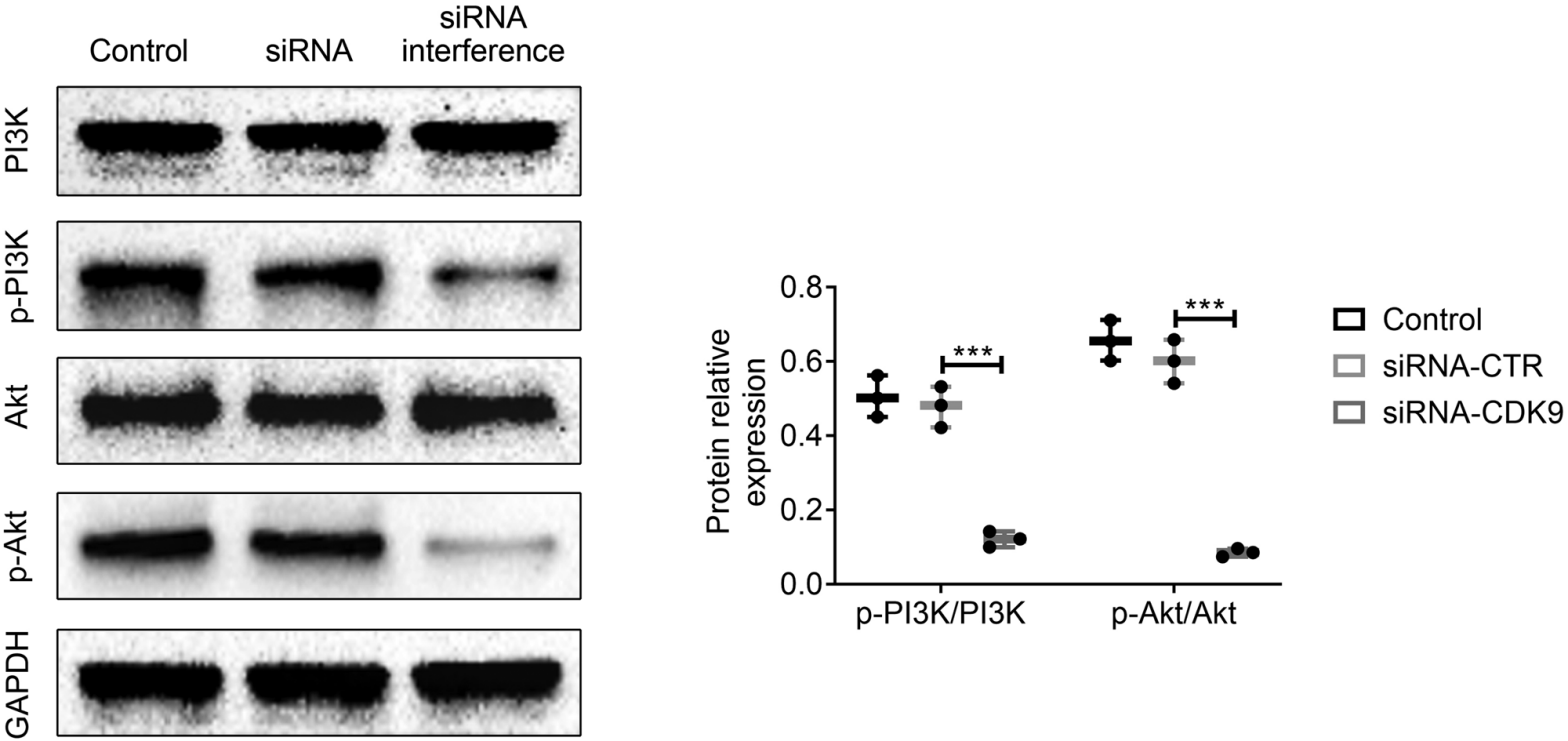
Effects of CDK9 interference on PI3K/AKT signaling pathway in HEC-1B cells. (a) Western blot assay was used to analyze the protein expressions of GAPDH, CDK9, p-PI3K, PI3K, p-AKT, and AKT in HEC-1B cells. (b) The protein relative expression levels of p-PI3K/PI3K and p-AKT/AKT from (a). Data were expressed as mean ± SD. ***p < 0.001 represents a statistically significant difference.

Western blot results showed that, compared with the siRNA-CTR and siRNA-CDK9 groups, the protein relative expressions of p-PI3K/PI3K and p-AKT/AKT in the siRNA interference group were significantly decreased (*p* < 0.05), as shown in Figure 5.

## 4 Discussion

Recent studies have shown that the high expression of CDKs could promote tumor development and generation of drug resistance, and the development of CDK inhibitors have been used to treat a variety of cancers [11,12]. CDK9 was, first, shown to play a significant role in human immunodeficiency virus transcription, and it has been shown to play a vital role in adenovirus, Epstein Barr virus, and human T cell lymphovirus infections in patients. In recent years, Kretz et al. [13] have shown that CDK9 is expressed in pancreatic cancer tissues, and the average overall survival of patients with high CDK9 expression group (10.92 months), which is lower than that of patients with low CDK9 expression group (20.59 months). Wang et al. [14] found that the expression level of CDK9 in metastatic and recurrent ovarian cancer tissues was significantly higher than that in tumor tissues with primary lesions, and the disease-free survival and total survival time of CDK9 positive group were significantly lower compared with that of CDK9 negative group. These results suggested that CDK9 might be an independent risk factor for cancer prognosis. In this study, CDK9 was found to be highly expressed in EC tissues, and its high expression was correlated with histological grade, FIGO stage, depth of muscular invasion, and lymph node metastasis. The 5 year survival rate of CDK9 positive group was lower than that of CDK9 negative group. These results suggested that CDK9 is upregulated during the development of EC, and its high expression is a factor for poor prognosis in EC.

Abnormal cell proliferation is a key factor leading to tumorigenesis, and the PI3K/AKT signaling pathway is involved in the proliferation of various cancer cells through the activation of downstream corresponding signaling pathways [15,16]. Current studies have shown that the PI3K/AKT pathway changes in 80% of EC patients, and rational drug design using molecular targets of PI3K/AKT signaling pathway is one of the important ways to treat tumors [17,18]. To investigate the biological role of CDK9 in EC, HEC-1B cells were transfected with CDK9 siRNA. MTT assay and plate colony formation assay showed that the proliferative ability of HEC-1B cells was significantly reduced, and the protein expressions of p-PI3K and p-AKT in the cells were also significantly reduced. It is suggested that CDK9 reduction may inhibit HEC-1B cell proliferation by inhibiting the activation of PI3K/AKT signaling pathway. This conclusion was supported by Xu et al. [8] and Lam et al. [19]. Xu et al. [8] has shown that CDK9 promotes tumorigenesis by a major role in activating the PI3K pathway. Lam et al. [19] showed that CDK9 regulates the tumorigenic activity of PI3K/AKT via the mitogen-activated protein kinase interacting kinase-eukaryotic initiation factor 4E axis.

In conclusion, the high expression of CDK9 has a close relationship with the occurrence and development of EC. It is thus inferred that CDK9 can be used as a new prognostic biomarker of EC. CDK9 siRNA significantly reduced EC cell proliferation by inhibiting the PI3K/AKT signaling pathway activation. More regulatory mechanisms need to be investigated in subsequent studies.
